# Evaluation of Acoustophoretic and Dielectrophoretic Forces for Droplet Injection in Droplet-Based Microfluidic Devices

**DOI:** 10.1021/acsomega.3c09881

**Published:** 2024-03-28

**Authors:** Jacqueline A. De Lora, Florian Aubermann, Christoph Frey, Timotheus Jahnke, Yuanzhen Wang, Sebastian Weber, Ilia Platzman, Joachim P. Spatz

**Affiliations:** † Department of Cellular Biophysics, Max Planck Institute for Medical Research, Jahnstraße 29, 69120 Heidelberg, Germany; ‡ Institute for Molecular Systems Engineering (IMSE), Heidelberg University, Im Neuenheimer Feld 225, 69120 Heidelberg, Germany; § Max Planck School Matter to Life, Jahnstraße 29, 69120 Heidelberg, Germany

## Abstract

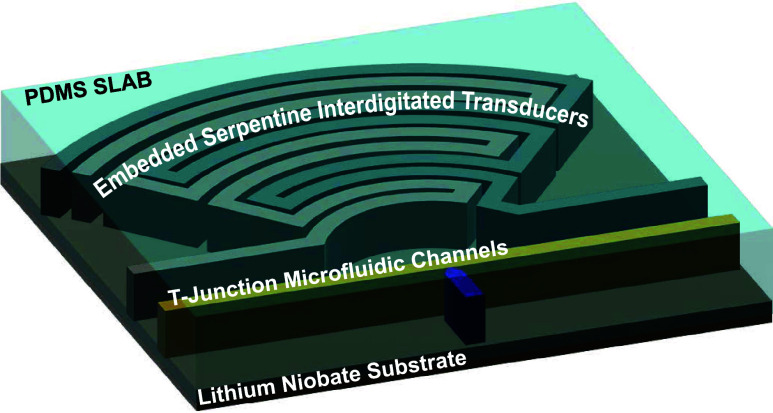

Acoustophoretic forces have been successfully implemented into droplet-based microfluidic devices to manipulate droplets. These acoustophoretic forces in droplet microfluidic devices are typically generated as in acoustofluidic devices through transducer actuation of a piezoelectric substrate such as lithium niobate (LiNbO_3_), which is inherently accompanied by the emergence of electrical fields. Understanding acoustophoretic versus dielectrophoretic forces produced by electrodes and transducers within active microfluidic devices is important for the optimization of device performance during design iterations. In this case study, we design microfluidic devices with a droplet injection module and report an experimental strategy to deduce the respective contribution of the acoustophoretic versus dielectrophoretic forces for the observed droplet injection. Our PDMS-based devices comprise a standard oil-in-water droplet-generating module connected to a T-junction injection module featuring actuating electrodes. We use two different electrode geometries produced within the same PDMS slab as the droplet production/injection channels by filling low-melting-point metal alloy into channels that template the electrode geometries. When these electrodes are constructed on LiNbO_3_ as the substrate, they have a dual function as a piezoelectric transducer, which we call embedded liquid metal interdigitated transducers (elmIDTs). To decipher the contribution of acoustophoretic versus dielectrophoretic forces, we build the same devices on either piezoelectric LiNbO_3_ or nonpiezo active glass substrates with different combinations of physical device characteristics (i.e., elmIDT geometry and alignment) and operate in a range of phase spaces (i.e., frequency, voltage, and transducer polarity). We characterize devices using techniques such as laser Doppler vibrometry (LDV) and infrared imaging, along with evaluating droplet injection for our series of device designs, constructions, and operating parameters. Although we find that LiNbO_3_ device designs generate acoustic fields, we demonstrate that droplet injection occurs only due to dielectrophoretic forces. We deduce that droplet injection is caused by the coupled dielectrophoretic forces arising from the operation of elmIDTs rather than by acoustophoretic forces for this specific device design. We arrive at this conclusion because equivalent droplet injection occurs without the presence of an acoustic field using the same electrode designs on nonpiezo active glass substrate devices. This work establishes a methodology to pinpoint the major contributing force of droplet manipulation in droplet-based acoustomicrofluidics.

## 1 Introduction

Active microfluidic devices that use kinetic stimuli such as acoustic or dielectric fields are increasingly being implemented.^1^ Such studies have exploited acoustophoresis for controllable manipulation of particles or cells within channels^2^ and, in one instance, combined the advantages of using both acoustic and dielectric forces to manipulate and sense cells.^3^ Scaling analyses for comparing the magnitude of acoustophoretic^4^ and dielectrophoretic^5^ forces on particles or cells with micrometer length scales further offer insights into the mechanisms at play in active microfluidic devices. Some numerical studies attempted to optimize acoustic force transmission,^6^ and in silico^7^ modeling has attempted to decouple the contribution of electrokinetic phenomena from acoustomechanical forces in acoustofluidic devices specifically for particle manipulation. While manipulation of particles and cells in suspension has been widely studied and demonstrates important applications in biomedicine,^8^ there are still opportunities to bring enhanced functionalities to the range of applications that droplet-based compartmentalization approaches enable especially with respect to controlled manipulations of different fluid compartments.

Devices that use kinetic stimuli to produce observable phenomena on or between water-in-oil droplets, regardless of the presence of particles or cells, can initially be divided into two categories based on design, materials, and operation. The first is droplet-based microfluidic devices that aim to achieve active functionalities such as droplet merging, splitting, deflection, or attraction within microfluidic channels using dielectrophoretic manipulation. For example, droplet-based microfluidic devices with electronic modules have introduced unique functionalities such as droplet sorting, feedback control modules, and picoinjection.^9−16^ In particular, picoinjection devices use an electric field to inject an aqueous phase into surfactant-stabilized water-in-oil droplets, passing a T-junction with an applied electric field. These devices use unique electrode structures that are themselves fluidic channels commonly filled with conductive metal solder and are, therefore, directly built into the same PDMS slab as the droplet channels. The electrode channels are then actuated by external electronics typically in the kHz frequencies at voltages above 300 V. The underlying mechanism for picoinjection, proposed by Abate et al.^15^ and supported by Herminghaus,^17^ is that the surfactant layer assembled at the interface between the droplet flowing over the picoinjection channel junction and the reagent in the pressurized picoinjection channel is destabilized by an electrically induced thin-film instability. This thin-film instability induced by the electric field is thought to physically cause spinodal dewetting of the surfactant layer at the interface between the droplet and the injection reagent, while the Laplace pressure law describes the observation of reagent entering the droplets as they pass by the junction. Spinodal dewetting of the surfactant layer induced by the electric field is on the nanometer-size scale in contrast to the forces that are required to manipulate particles or cells on the micron scale. The second category extends the idea of manipulating droplets with kinetic stimuli to the field of acoustofluidics, where devices with complex functionalities have demonstrated acoustic droplet production, merging, splitting, and mixing.^18−27^ To achieve such droplet manipulations, electrodes are typically patterned onto a piezoelectric wafer with an interdigitated transducer (IDT) geometry that is open to the air and aligned to an independent PDMS slab containing either channels or open chambers. The IDTs are aligned to the propagation axis of the piezoelectric substrate, and the PDMS channels are oriented to exploit acoustophoretic forces. During operation, an amplified electronic waveform, usually in MHz frequencies and in 0.2–3 V ranges, is broadcast along the active and passive finger pairs of the IDTs. These then actuate the underlying piezoelectric substrate, thereby converting electrical energy into acoustic waves. Depending on the geometry, material, and electromechanical coupling coefficient of the IDTs and substrate, the type of acoustic wave propagation and the planes in which it travels in the device will lead to different acoustophoretic forces.^28^ The distance between the finger pairs and degree of serpentine geometry determines the resonance frequency, and finally, the input power to the IDT as well as height considerably influences the resonance frequency and amplitude of the resulting acoustic field.^29^

As many device-production techniques overlap between active droplet-based microfluidics and acoustofluidics, it is not surprising that device designs combining techniques from both fields to simplify construction are being explored. For example, patterning electrodes as in standard acoustofluidics is more labor-intensive and costly than producing standard electric-field-mediated picoinjection devices that use the embedding technique. Therefore, some researchers took the rational next design step to apply the electrode-embedding technique from standard picoinjection units to acoustofluidic devices. In this approach, IDTs are fabricated just as with microfluidic devices with embedded electric units, where a conductive metal is filled within fluidic channels on the same PDMS slab as the droplet microfluidic channels. The main difference here is that the substrate is piezoelectric rather than glass, turning the electrodes into transducers. While this approach offers direct control over IDT geometries and fluidic channel alignment, bonding of PDMS to the piezoelectric substrate, usually lithium niobate (LiNbO_3_), can be a challenge due to different surface chemistries compared to glass. This bonding challenge can result in channels delaminating when under pressure or that require less common oxygen and nitrogen plasma treatments to achieve a sufficient bond between the LiNbO_3_ and PDMS.^30^ Regardless, embedded IDT devices for acoustofluidic applications have so far been implemented using surface acoustic waves (SAWs) at MHz resonant frequencies and with up to 50 V of power for droplet production^31^ and mixing within a single aqueous droplet^32^ as well as within a channel separated from the IDTs with an air gap.^33^ It is worth noting that Nam et al.^33^ suggest the necessity for low conductivity fluids and >300 V to generate appreciable electrokinetic forces in acoustofluidic devices with embedded liquid metal IDT architectures generating SAWs. Finally, SAWs have been experimentally and theoretically described to influence fluids with nanoscale features at interfaces, including to cause morphological instabilities.^34−37^ On the other hand, bulk acoustic pressure waves are understood to function well for manipulating particles and cells on the micrometer scale as the wavelength for manipulation scales with the particle size. Pressure waves generally do not function at nanoscale lengths to cause phenomena such as thin-film instability due to gaps in time scales, making it difficult to derive analytical solutions.^38^

The convergence of droplet-based microfluidics and acoustofluidics led us to the idea of combining aspects of droplet picoinjection device constructions such as the T-junction injection module and relatively simple production of embedded liquid metal interdigitated transducers (elmIDTs) with components of acoustofluidic devices such as transducer geometry and the piezo-active substrate LiNbO_3_. We envisioned producing a device that would enable acoustic field-mediated injection of droplets to explore a parameter space that would potentially enable precise volumetric injection of reagents, particles, and cells into droplets that would be amenable to further downstream analysis methods and applications.

While we present device construction with PDMS-embedded curved serpentine-paired IDTs that indeed combines droplet generation and injection modules on one chip, we demonstrate that our hypothesis for acoustically actuated droplet injection is null. We visualize the generation of acoustic field geometries within our devices; however, we find that the operating parameters where we observe droplet injection support a mechanism of droplet injection by dielectrophoretic forces. As such, we report here a practical method to deduce the mechanism for droplet injection on either piezoelectric (LiNbO_3_) or nonpiezoelectric (glass) substrates with elmIDTs or PDMS-embedded picoinjection electrode geometries (PI), operated at a range of radio frequencies, electrode polarity, alignments, and voltages. Our methodology provides an experimental design strategy that can be used to validate the source of kinetic stimuli in microfluidic devices with electronics-based functional modules and can further inform in silico modeling to predict and understand observable phenomena.

## 2 Results and Discussion

### 2.1 Structure and Harmonic Wavelength of the Device

**Figure 1:**
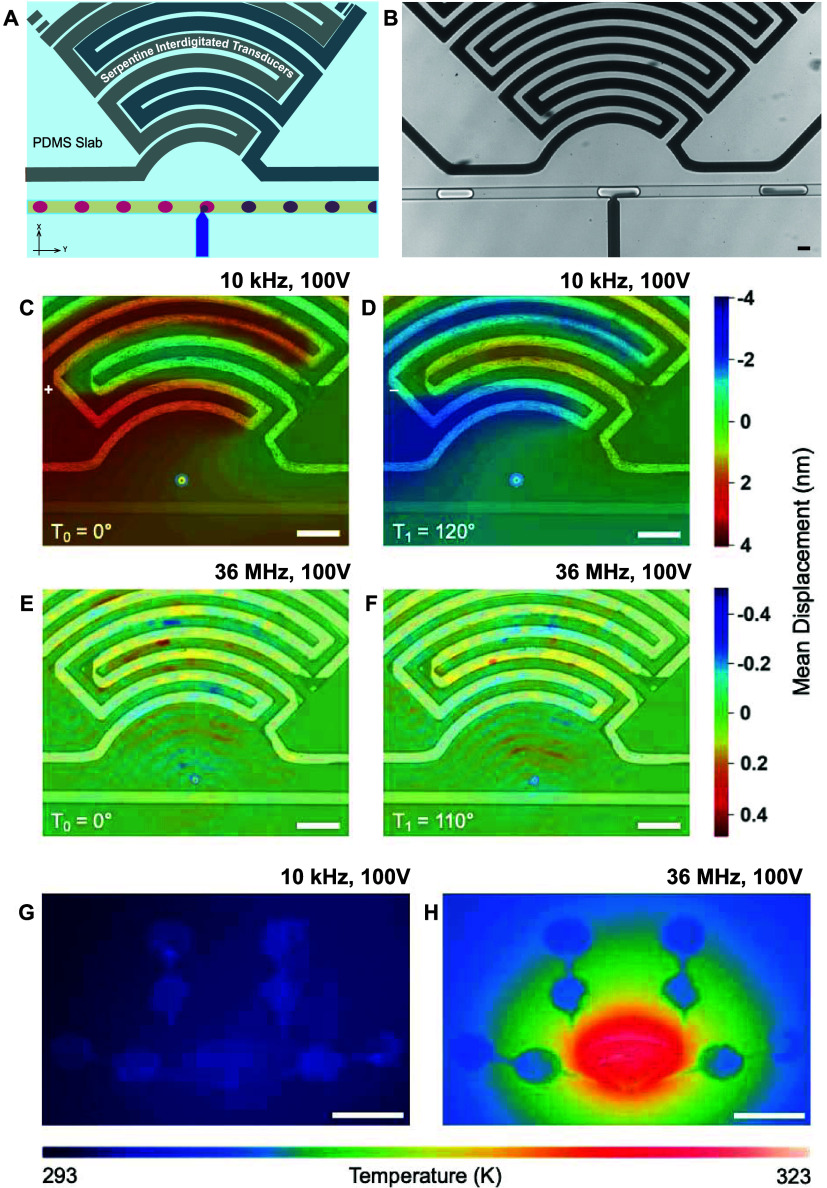
Characterization of a droplet-based injection device: (A) Illustration of the manipulation area of the device. At the T-Junction, the surfactant-stabilized interface of the passing droplets (pink) is disrupted, enabling the injection of the fluid phase of the injection channel (purple). (B) Bright-field image of the device demonstrating the injection into a surfactant-stabilized water-in-oil droplet. Scale bar: 30 μm. (C–F) Microscopy-based laser Doppler vibrometry (LDV) measurements of the device on LiNbO3 from two frequency domains at time points that attribute to different amplitudes (1 Amplitude: Tπ = 180°; LDV displacement in the range: 0–180). (C, D) LDV measurements obtained while operating the device at 10 kHz and 100 V. The form of the acoustic field, visualized by the color map overlay of the mean displacement field, demonstrates a pressure wave with displacements in the nanometer range. (E, F) Device operating at 36 MHz and 100 V with the mean displacement mapping showing the generation of a focusing SAW with an order of magnitude lower displacement. Scale bars: 100 μm. (G, H) Infrared images of the device on LiNbO_3_ after operating for 6 s at two frequencies of interest. For panel (G), the frequency is set to 10 kHz, the input voltage is set to 100 V, and the thermal increase is negligible. For panel (H), the frequency is set to 36 MHz and the input voltage to 100 V. In the high-frequency range, there is a thermal increase up to 30 K in the region of the IDT. Scale bars: 2 mm.

The device comprises either a glass (nonpiezoelectric) substrate or an optical-grade piezoelectric 128° Y-cut LiNbO_3_ substrate containing a microfluidic droplet generation module, T-junction injection channels, as well as focused, serpentine, and elmIDTs integrated into one PDMS slab. Figure 1A illustrates our design with the orientation of the elmIDTs and fluidic channels in the propagation direction for the LiNbO_3_ wafer, and Figures S1 and S2 present the blue-prints including dimensions of continuous flow acoustofluidic and picoinjection microfluidic devices used with explanations for the functions of the inlets and outlets. The orientation of the channels on the glass substrates does not play a role. Figure 1B shows a frame from a high-speed camera video obtained with a brightfield microscope. The image demonstrates an approaching empty droplet (left), a droplet in the process of being injected (center), and an injected droplet flowing toward the collection port of the device.

The device uses an elmIDT with a curved serpentine geometry, where each finger pair reduces in radii until a particular aperture diameter is achieved. Our design comprises 10 finger pairs, and we hypothesize that it will focus the harmonic wavelength to the injection nozzle region of the flow channels. The harmonic wavelength is described by eq 1, adapted from^33^
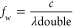
1where *f*_w_ is the resonant frequency, *c* is the propagation speed of sound in LiNbO_3_ (approximated to be 3700 m/s), and λ double is the harmonic wavelength for a double-electrode IDT (8 times the width of the electrode channel). Given our design of 30 μm wide channels, a harmonic excitation frequency of 15 MHz is calculated. This does not take into account the 30 μm height dimension of the 10 finger-paired electrodes that are embedded into the PDMS as with picoinjection devices, which could also contribute to observed mismatches between the calculated and experimentally measured resonance.

When operating our device for the first time, we did not observe acoustic manipulations of water-in-oil droplets through a sweep of MHz excitation frequencies. During subsequent empirical testing using frequency sweeps across a broader range of domains, we surprisingly observe droplet manipulations in the audible frequency domain (kHz ranges), 3 orders of magnitude lower than the expected resonant frequency. We then set out to investigate why droplet manipulation in the kHz range is possible and to understand the influence of PDMS, channel geometries, and elmIDTs.

### 2.2 Characterization of Acoustophoretic Forces

Visualization of the acoustic fields produced by our devices on LiNbO_3_ in the empirically determined kHz frequency domain as well as at a sweep of MHz frequencies is essential to understand acoustophoretic forces generated. The corresponding characteristics of the acoustic fields, including field geometry, mean displacements caused by the transduced oscillations, and thermal contributions, are also fundamental to understanding the discrepancy of our system with design guidelines. We perform LDV, Figure 1C–F, and infrared imaging measurements, Figure 1G,H, providing both visual and physical understanding of the acoustophoretic forces. LDV enables visualization of the acoustic field shape, for example, pressure waves vs standing waves, by measuring the magnitude of acoustically induced displacements propagating through the system. We obtain LDV measurements and present representative heat maps that show resonant peaks of interest along with the mean displacements measured within the injection region of the devices. At 10 kHz with 100 V, we observed a bulk acoustic pressure wave with mean displacements in the nanometer range (Figure 1C,D). As seen in Video S1, an alternating deflection of the respective finger pairs causes a pressure wave across the injection nozzle region of our device. Table S1 lists the respective values for the displacements across the set of operating parameters investigated. In contrast to this, we do indeed observe a focused SAW propagating through the system (Video S2) when operating the device near the theoretical resonance; however, we identify a stronger field at 36 MHz and 100 V, a mismatch arising due to assumptions made when calculating the harmonic wavelength for hard-metal electrodes. The acoustic wave focuses to the injection nozzle region of our device, however, with a notable decrease in the mean displacement (Figure 1E,F and Table S1). We expect that the discrepancy between our physical observations in the audible frequency range in contrast with MHz frequencies is caused by embedding the IDTs with matched channel heights into the PDMS slab. For example, other researchers have observed significant damping of SAWs when directly overlying PDMS onto hard-metal IDTs patterned on LiNbO_3_ substrates.^39,40^

Considering the transmissivity gained by embedding the IDTs and our empirical observation of droplet injection at kHz frequencies, we then evaluate damping or thermoviscous contributions. We use infrared imaging to visualize thermal excitations using the same conditions measured by LDV. When the device is operated in the pressure wave mode at 10 kHz and 100 V, we observe minimal increases in thermal energy (Figure 1G). In comparison, at 36 MHz and 100 V, the parameter combination where our device generates strong SAWs, the temperature in the device drastically increases to 323 K (Figure 1H). This is in agreement with other devices whereby SAWs applied across an interface are thought to refract into the fluid, leading to compressional waves that may enable acoustic streaming within droplets. The refraction of SAWs at such interfaces, especially with conventional IDTs open to air, is usually associated with attenuation of the waveform amplitude and generation of heat. In particular, the thermoviscous contribution in operating acoustofluidic devices at MHz frequencies has been shown to evaporate fluids in digital microfluidics and has negative impacts on the rheological properties of suspension media, in vitro protein stability, and cell viability.^41,42^ For example, many systems that operate in the MHz frequency domain restrict exposure time of biological samples to the acoustic field^43^ or implement feedback temperature control strategies, including Peltier stages^44^ to mitigate thermal contributions. Other works attempt to exploit device heating by using the phenomena in applications such as continuous flow polymerase chain reactions^39^ or to deliberately kill cells.^23^

Taken together, our measurements and literature validate that in our system, the density of the PDMS and speed of sound leads to the refraction of MHz-generated SAWs into the PDMS layer, thereby developing heat. The diminished displacement coupled with thermoviscous losses in the MHz frequency domain attenuates the acoustophoretic force of the generated SAWs. Therefore, at the resonant frequency, the surfactant shell at the interface between water-in-oil droplets as they pass the injection nozzle is not destabilized. This leads us to question if there might also be dielectrophoretic contributions from the elmIDTs as this is the typical operating parameter space for canonical microfluidic picoinjection on glass substrates and could explain why we observe droplet injection at kHz frequency modulations.

### 2.3 Deciphering the Forces Underlying Droplet Injection

**Figure 2:**
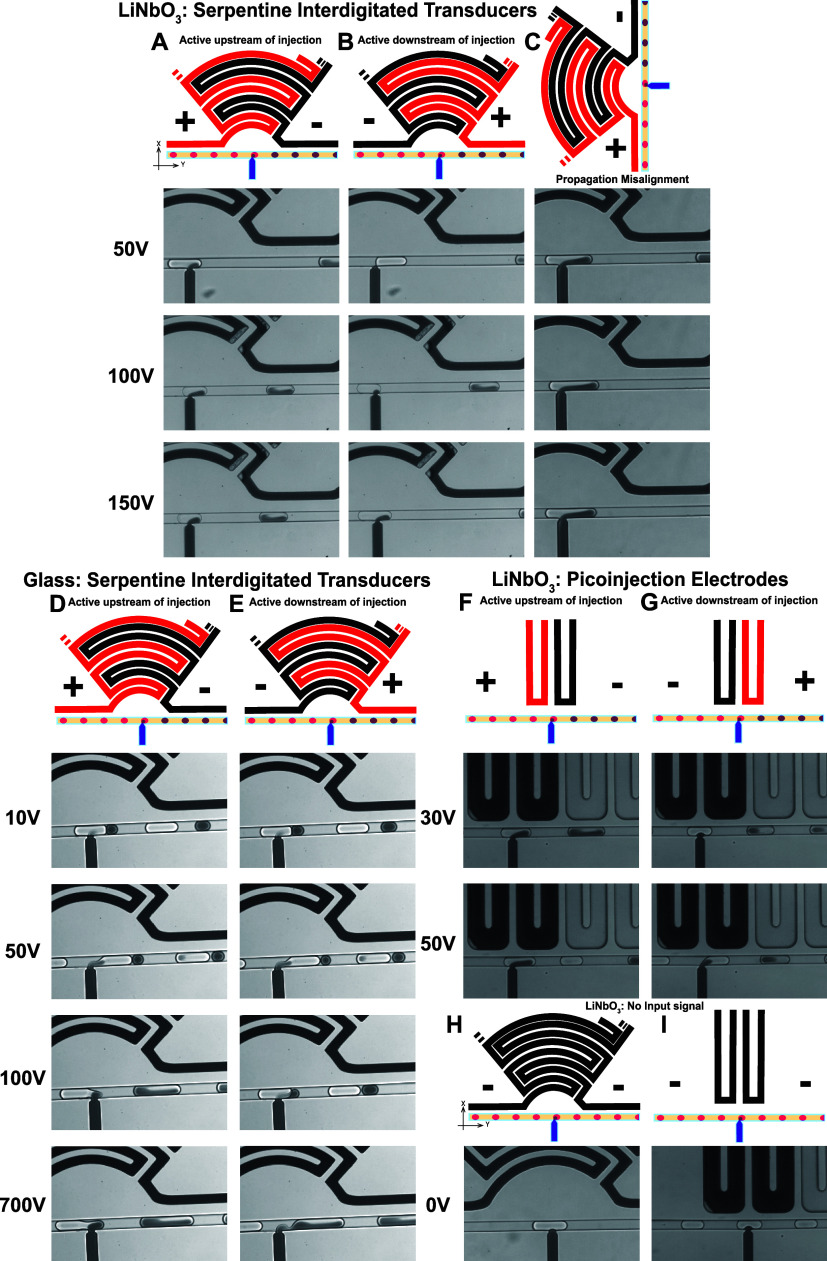
Acoustophoretic vs dielectrophoretic contributions for manipulating droplets in injection devices. (A–C) Image frames from bright-field high-speed camera videos demonstrating devices constructed onto piezoelectrically active LiNbO_3_ substrates with elmIDTs. Columns (A, B) are constructed with the elmIDTs aligned with the propagation direction of the piezoelectric wafer, whereas (C) is misaligned by 90° to the wafer notch. Further, the devices are operated with the active electrode (red in the illustrations) either upstream (A, C) or downstream (B) of the injection region, using 50, 100, and 150 V of power as indicated for each row. (D, E) Image frames using the exact same device design as (A–C) except constructed on glass, a nonpiezo-active substrate. Additionally, the electrode polarity is active upstream in (D) or downstream in (E) at 10, 50, 100, and 700 V. (F, G) Image frames of devices built with canonical PI electrodes on LiNbO_3_ substrates, operated with upstream (F) and downstream (G) electrode polarities at 30 and 50 V. As a final control demonstration, panels (H, I) show image frames of droplets flowing over the T-junction of the channels without an input signal to the two different electrode configurations, showing no droplet injection.

To delineate whether droplet injection in our device design is attributed to acoustophoretic or dielectrophoretic forces, we build up a series of device constructions where the impact of each force can be evaluated as an independent variable when droplet injection is observed. First, we construct the acoustofluidic device on to LiNbO_3_ as for the characterizations addressed in Figure 1, adding a droplet production module with an extended winding geometry such that the droplets are sufficiently stabilized by the surfactant before reaching the injection T-junction. Each device is operated with a slightly varying flow rate for the droplet production to operate stably; however, the injection pressure from the orthogonal channel of the T-junction is kept consistent at 140 mbar. The device is constructed in two orientations: (1) with the elmIDTs aligned to the propagation direction of the wafer (Figure 2A,B) and (2) with the elmIDTs misaligned or rotated 90° to the propagation direction of the wafer (Figure 2C). All device elmIDTs are operated at 10 kHz with either 50, 100, or 150 V. Finally, in the first instance of proper elmIDT alignment, the electrodes are connected with the polarity such that the active electrode (red) is either upstream or downstream of the T-junction, as illustrated in Figure 2A,B, respectively. We observe that the magnitude of the volume injected into the droplets changes with increasing voltage and reversing the polarity of the electrodes, especially at 50 V, attenuates the volume of the injected fluid but is recovered at higher power. Importantly though, since the speed of sound in LiNbO_3_ is dependent on the elmIDT orientation with respect to the wafer, if acoustophoretic forces alone are underlying droplet injection, we expect to at least diminish droplet injection for the second device construction described. We observe that the devices constructed with the elmIDTs misaligned to the propagation direction of the wafer, Figure 2C, still demonstrate injection at all applied voltages. This finding leads us to hypothesize that acoustophoretic forces may not be the only forces at play to mediate droplet injection.

To test the presence of dielectrophoretic forces in our device design and evaluate the dependence on power, we then construct the exact same devices onto glass, a nonpiezoelectrically active substrate. Similarly to the devices constructed on LiNbO_3_, we operate the elmIDTs with the active electrode (red) upstream (Figure 2D) vs (black) downstream (Figure 2E) of the injection T-junction at 10 kHz with 10, 50, 100, and 700 V. Each parameter combination, except for the lowest power/reversed electrode polarity condition, demonstrates droplet injection. However, the accuracy of the injection is not as consistent as when the devices are constructed onto LiNbO_3_ suggesting that there may be an influence by the acoustic field. This leads us to then evaluate if the geometry of the elmIDTs has an impact on the injection reproducibility by using a canonical PI device constructed on to LiNbO_3_. We find that at lower voltages than normally used for PI, 300 V as the lower end bench mark as suggested by Nam et al.,^33^ we are able to achieve picoinjection of droplets using a typical PI electrode geometry (Figure 2F) and that it is somewhat independent of the electrode polarity (Figure 2G). Finally, no droplet injection is observed in the absence of an input signal, demonstrating that the T-junction geometry alone is not sufficient to cause droplet manipulations (Figure 2H,I).

Taken together, we ultimately find that droplet injection observed at kHz frequencies in our device series constructed on piezo-active substrates can not only be attributed to the acoustic field. This is because (1) we only observe droplet injection at kHz frequencies where the acoustic field geometry is a pressure wave rather than a SAW; (2) we observe droplet injection on LiNbO_3_ even when the IDTs are misaligned with the propagation direction of the wafer; and (3) we observe droplet injection on nonpiezoelectric substrates using the same elmIDTs at kHz frequencies. In another way, our experiments do not comprehensively decouple the acoustic field from the electric field but rather demonstrate that equivalent droplet injection occurs without the presence of an acoustic field. This suggests that the contribution from the acoustic field in our specific devices is negligible and that dielectrophoresis describes the mechanism of droplet injection.

## 3 Conclusions

In this study, we present an empirical and practical strategy for understanding the impact of coupled acoustophoretic and dielectrophoretic forces within droplet-based microfluidic injection devices. We demonstrate one-chip microfluidic devices that enable the production and injection of water-in-oil droplets at kHz frequencies and believe that for this particular design, the observed droplet injection is likely not mediated solely by acoustophoresis as we originally hypothesized. Devices constructed on LiNbO_3_ substrates, where an acoustic field can be produced, function to manipulate water-in-oil droplets at kHz frequencies. This is in contrast with the theoretically predicted MHz resonance frequency, which is characteristic for the generation of SAWs within LiNbO_3_ devices. Using LDV measurements, we visualize a precisely focused SAW at MHz frequencies. However, infrared camera measurements clearly demonstrate that at these MHz frequencies, the energy that could otherwise cause droplet manipulations is lost as heat. While there is a SAW generated at frequencies at or near the predicted resonance frequency, these observed thermoviscous losses offer an explanation for our observation of a lack in droplet injection in the MHz frequency domain.

When operating at kHz frequencies, our device transmits an acoustic pressure wave with minimal thermal losses. However, the mismatch with the predicted resonance frequency led us to consider if then dielectrophoretic forces may also have an impact on observed droplet injections. We demonstrate a collection of experiments in which we attempt to delineate the respective contribution of the acoustophoretic versus the dielectrophoretic forces that cause the droplet injection. To this end, we use an experimental matrix of different device constructions on either non- or piezoelectric substrates with a range of operating parameters. We conclude that the contribution of dielectrophoretic forces should be considered with IDTs built into the same PDMS slab as microfluidic channels, especially when they are constructed on piezoelectric substrates without air gaps and even operated with voltages below 300 V.

To our knowledge, this work is the first to provide a rational and empirical methodology to address the source of kinetic stimuli produced within microfluidic devices with electronic modules. Our findings serve as a practical guide for researchers testing similar devices in attempting to produce an acoustofluidic device with elmIDTs for acoustic-field-mediated fluid manipulations. In the future, the acoustomechanical coupling coefficient could be optimized to improve the functioning of our design, and a numerical simulation could further elicit the decoupling of the forces from the inherently linked electric and acoustic fields generated by IDTs, but that is out of the scope of the current report. Further, our findings may be of use to those researchers engaged with in silico modeling of acoustofluidic devices as well as microfluidic devices with integrated electronic units, where the goal of predicting the discrete contributions from either acoustophoretic or dielectrophoretic forces is attempted. Our characterization method is worth serious consideration as a strategy to mechanistically validate observed interface and droplet manipulations in subsequent continuous flow and droplet-based acoustofluidic experiments.

## 4 Materials and Methods

### 4.1 Device Production

The acoustofluidic device is designed with computer-aided design (CAD) software, QCAD-pro (Ribbonsoft, Switzerland), and transferred onto a photoresist-layered silicon wafer (master wafer) with a micro Pattern Generator μPG 101 (Heidelberg Instruments, Germany). Figures 1, S1, and S2 show our designs and explain the constructions. As previously described,^12^ for the production of the master wafer, negative photoresist (SU8-3025, MicroChem, USA) is spin-coated (Laurell Technologies Corp., USA) onto a silicon wafer at 2650 rpm to achieve a uniform coating of 30 μm thickness. The wafer is then placed on a hot plate for a 5 min soft bake at 65 °C, and then ramped slowly to 95 °C and held for 15 min. The CAD design is exposed onto the photoresist with the writing mode II setting of the micro Pattern Generator. The output power of the laser is set to 50 mW with a pixel pulse duration of 20%. For the post-exposure bake, the wafer is placed on a hot plate for 1 min at 65 °C and then ramped and held at 95 °C for 5 min. The unexposed parts of the resist are removed with mr-DEV 600 (MicroChemicals, Germany). The following hard bake is carried out in an oven at 150 °C for 15 min.

To fabricate all of the acoustofluidic devices and PI devices on LiNbO_3_ or glass, polydimethylsiloxane (PDMS) (Sylgard 184, Dow Corning, USA) is mixed at a 9:1 (w/w) ratio and poured over the master wafer, degassed for several minutes in a desiccator, and cross-linked for 2 h at 70 °C in an oven. After hardening, the PDMS slab is peeled off of the wafer, and a biopsy punch is used (World Precision Instruments, USA) to punch holes for the fluid inlets/outlets (0.5 mm) and electrodes (1.5 mm). Prior to the attachment of the PDMS to either (1) a 2-propanol cleaned, 2-inch, double-side polished, lithium niobate (LiNbO_3_) piezoelectric crystal (128° Y-cut, 0.25 mm, Precision Micro-Optics, USA) or (2) a 60 mm round, 1.0 cover glass (Carl Roth GmbH, Germany), the PDMS and wafer are activated using an oxygen plasma (PVA TePla 100, PVA TePla, Germany; 0.5 mbar, 250 W, 5 min for the wafer followed by an additional 30 s with the PDMS). After activation of the LiNbO_3_ wafer, we found that adding 20 μL of Milli-Q water near the notch of the LiNbO_3_ wafer and then placing the PDMS slab with proper IDT alignment and light pressure on to the substrate followed by heating for at least 24 h at 80 °C achieve bonding. The IDT/PI channels are directly integrated into the microfluidic design and subsequently filled with liquid metal. For this, the microfluidic device is heated to 80 °C on a hot plate, and a low melting-point alloy (In_0.51_Bi_0.325_Sn_0.165_, Indium Corporation of America, USA) is molten injected into the IDT or PI channels. Electric wires are connected to the melted solder and fixed with UV hardening glue (Loctite 352, Henkel, Germany) after allowing the molten solder to cool down and harden.

### 4.2 Electronic Setup

Because our device operates across a broad-spectrum frequency range that includes both kHz and MHz frequencies, we use two different electronic set-ups to create and control the electronic sine waveforms applied to the embedded IDTs. In the range of 1–50 kHz, we create the sine waveform using an arbitrary function generator (Rohde & Schwarz, model HM8150, Germany) across a range of applied peak-to-peak power that is then amplified by a factor of 100 using a high voltage amplifier (TREK Model 2210, Acal BFi, Germany). In the MHz range, we create the sine waveform using an arbitrary function generator (AFG1062, Tektronix, Germany) with the same range of applied peak-to-peak power, amplified by a custom-built amplifier comprising a Mini-Circuits Model ZHL-32A+ op-amp powered by an RS-PRO RS-3005D Digital Control DC Power Supply. All electronic connections are established using standard BNC cords, connectors, and test clip adapters from Thorlabs Inc.

### 4.3 Device Characterization

#### 4.3.1 LDV

We use LDV to evaluate the contribution of acoustic energy to surface topography and dynamic motion as well as to visualize the operation of our acoustofluidic device. Our devices are evaluated using a full-field scanning microscope-based vibrometer (MSA-600 X/U, Polytec GmbH, Germany) and software (PSV, Polytec GmbH, Germany) that enable us to measure subnanometer displacement of our PDMS-embedded IDTs as a function of modulation across frequency and amplitude parameters. To record acoustic displacement maps, we use an opaque device that is suspended on an observation stage, such that only the edges of the SAW wafer are in contact with the mount. We then obtain scans of at least one full elmIDT finger pair along with the region where the T-junction of our microfluidic channels reside using combinations of frequencies and applied voltages. These bandwidth scans allowed us to identify potential resonance frequencies of our devices (10, 20, 30, 50, 5, 21, and 36 MHz) at a range of applied voltages (50, 100, and 150 V).

#### 4.3.2 Infrared Imaging

The infrared image measurements are taken with an IRCAM EQUUS 327k M (IRCAM, Germany) infrared camera at a wavelength of 3.7–5 μm, a 640 × 512 px resolution, a 15 μm pitch size, and a NETD < 20 mK. The thermographic images are recorded with a 100 Hz Framerate.

#### 4.3.3 IDT Amplitude Calibration and Influence of Different Devices

Three devices were built to measure the impact of the PDMS slab positioning and elmIDT quality on the piezoelectric effect propagating through the LiNbO_3_ substrate. A sine wave of variable amplitude between 0.5 and 5 V at 10 kHz was fed to the amplifier, and the resulting peak-to-peak voltages were measured across the IDTs. Measurements were performed using an oscilloscope (Voltcraft DSO-1062D 2 Channel Digital Storage, Germany) using a standard BNC cord connected to a 100× probe.

### 4.4 Droplet Microfluidics and Injection

#### 4.4.1 Production of Water-in-oil Droplets

We produce surfactant-stabilized water-in-oil droplets using the flow-focusing junction module of either the acoustofluidic or PI devices (Figures S1 and S2). The continuous phase comprises a 3 wt % perfluoropolyether–poly(ethylene glycol) (PFPE–PEG) block copolymer fluorosurfactant (Ran Biotechnologies, Inc., USA) dissolved in fluorinated oil (HFE-7500, 3M, USA). The dispersed phase is pure Milli-Q water. Both phases are injected into the production module with our pneumatic flow controller (Elveflow, Microfluidic Flow Controller model OB1 3+, France) that connects 1.5 mL Eppendorf tubes to the fluidic inlets and outlets of the PDMS slab using PTFE tubing (1/32″ ID, Elveflow, France).

#### 4.4.2 Device Operation for Injection

Each experiment is initiated by mounting the device onto our inverted microscope (Zeiss AxioVert 200, Germany) and optimizing the parameters for imaging with our high-speed camera (Phantom v7_3, Vision Research, USA). The electrodes are connected in the desired orientation to the electronic setup described in Section 4.2. Then, we optimize droplet generation on the chip using the upstream droplet production module of the devices by varying the droplet inlet pressures for both oil and water phases between 100 and 400 mbar. Meanwhile, the injection channel is pressurized to around 110 mbar to prevent backflow of oil into the injection channel. Once a steady droplet production rate is achieved, we then adjust the pressure in the injection fluid channel to 140 mbar for the experiments, although we find that a range of injection pressures from 120 to 170 mbar is possible. For better visualization, black ink is used as the droplet injection fluid. Then, we adjust the parameters for the desired electronic input signal and coordinate the capture of high-speed camera videos with the electrodes in their active state.

## Supporting Information Available

Additional LDV measurements and device blue-prints, including LDV of devices operated at different frequencies (PDF)
LDV video, 10 kHz frequency, 1 V_p–p_ input amplitude, and pressure wave (MP4)
LDV video, 36 MHz frequency, 1 V_p–p_ input amplitude, and SAW (MP4)
3 wt % droplet injection, 10 MHz frequency, and 3 V_p–p_ input amplitude (AVI)

## Supplementary Material

ao3c09881_si_003.mp4

ao3c09881_si_004.avi
