# Supplementary material for: Evaluation of Acoustophoretic and Dielectrophoretic Forces for Droplet Injection in Droplet-Based Microfluidic Devices
Source: ACS Omega. 2024 Mar 28;9(14):16097–105. doi: 10.1021/acsomega.3c09881 (PMC11007716; doi:10.1021/acsomega.3c09881)
Supplement: Supplementary file 1 — ao3c09881_si_001.pdf [file ao3c09881_si_001.pdf]

Supplementary information

Evaluation of acoustophoretic and dielectrophoretic forces for droplet injection in droplet-based microfluidic devices

Jacqueline A. De Lora,[ab#*] Florian Aubermann,[abc#] Christoph Frey,[ab#] Timotheus Jahnke,[ab] Yuanzhen Wang,[ab] Sebastian Weber,[ab] Ilia Platzman,*[ab] Joachim P. Spatz*[ab]

[a] Department of Cellular Biophysics, Max Planck Institute for Medical Research, Jahnstraße 29, 69120 Heidelberg, Germany.

[b] Institute for Molecular Systems Engineering (IMSE), Heidelberg University, Im Neuenheimer Feld 225, 69120 Heidelberg, Germany.

[c] Max Planck School Matter to Life, Jahnstraße 29, 69120 Heidelberg, Germany.

# Authors Contributed Equally
*Corresponding Authors: jacqueline.delora@mr.mpg.de (J.A. De Lora), ilia.platzman@mr.mpg.de (I. Platzman), and joachim.spatz@mr.mpg.de (J.P. Spatz)

This file includes

Table S1: LDV measured mean acoustic displacements.
Figure S1: Acoustofluidics device blue-print.
Figure S2: Picoinjection device blue-print.

Other supplementary materials:
Video S1: LDV video, 10 kHz frequency, 1 V_{p-p} input amplitude, pressure wave
Video S2: LDV video, 36 MHz frequency, 1 V_{p-p} input amplitude, SAW
Video S3: 3 wt% Droplet injection, 10 MHz frequency, 3 V_{p-p} input amplitude

Table S1: LDV measurements of mean acoustic displacements.

Frequency	Applied Voltage (V_{p-p})	Displacement (nm)
10 kHz	0.5	0.875
	1.0	1.717
	1.5	2.526
20 kHz	0.5	0.013
	1.0	0.029
	1.5	0.030
30 kHz	0.5	0.091
	1.0	0.089
	1.5	0.173
50 kHz	0.5	0.104
	1.0	0.256
	1.5	-
5 MHz	1.5	0.002
21 MHz	1.5	0.002
36 MHz	1.5	0.087

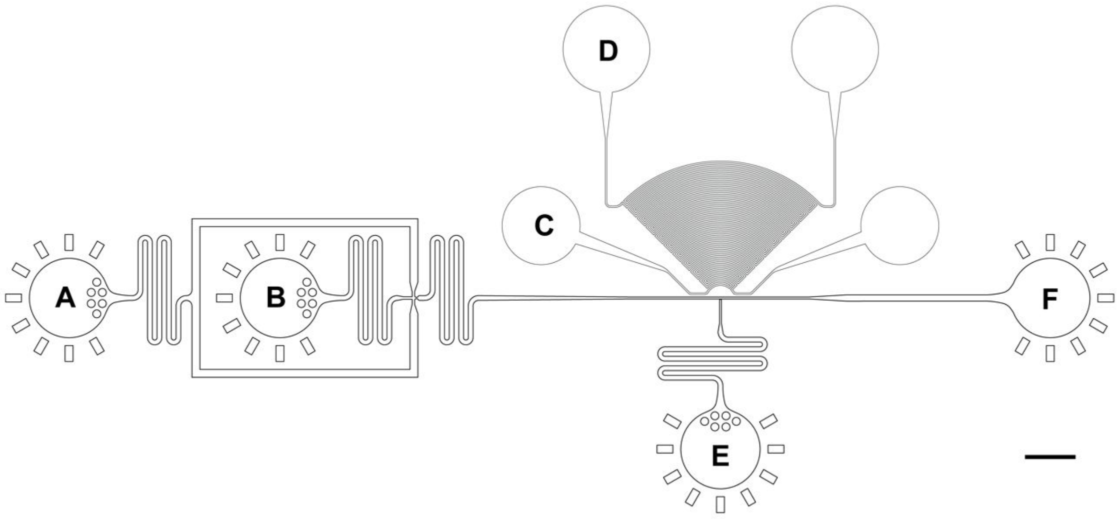

Figure S1 Acoustofluidics device blue-print. **(A)** Injection channel for oil phase of droplet production module. **(B)** Injection channel for water phase of droplet production module. **(C)** Injection channel for liquid metal that will fill the upstream electrode of the elmIDT. The mirroring injection channel (unlabelled) is likewise, the injection channel for liquid metal that will fill the downstream electrode. **(D)** Port for electrode contacts to wires and likewise for the unlabelled downstream electrodes. **(E)** Injection channel for injection fluid in the T-junction. **(F)** Outport for droplet collection. Scale bar is 1 mm.

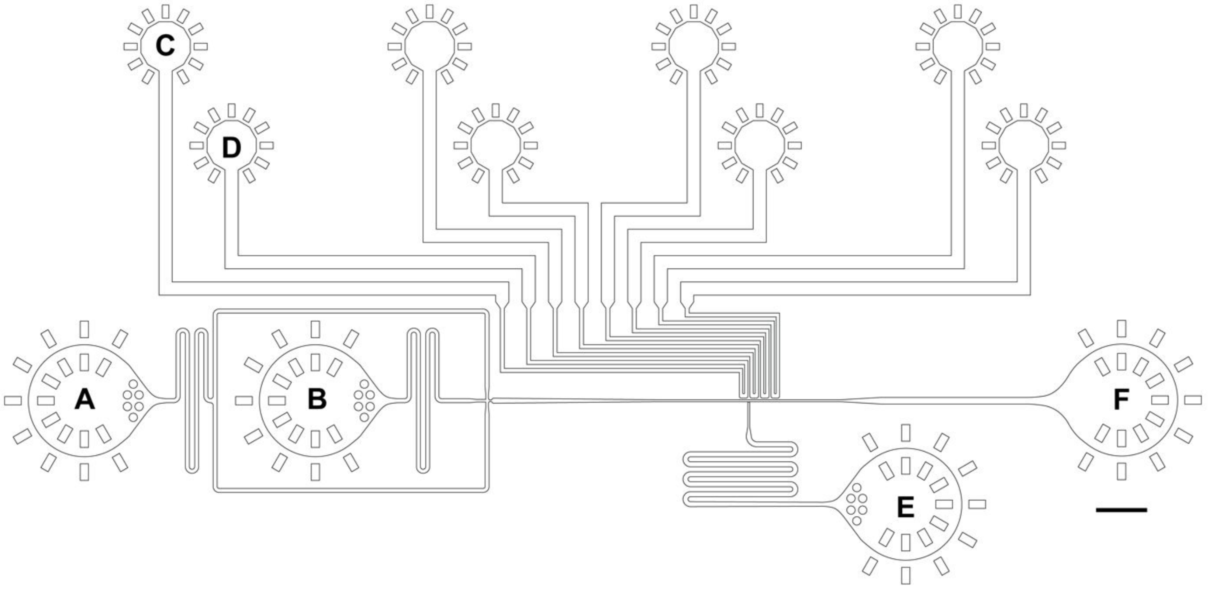

Figure S2 Picoinjection device blue-print. **(A)** Injection channel for oil phase of droplet production module. **(B)** Injection channel for water phase of droplet production module. **(C)** Injection channel for liquid metal that will fill the upstream PI electrode. The mirroring injection channel (unlabelled) is likewise, the injection channel for liquid metal that will fill the downstream electrode. **(D)** Port for electrode contacts to wires and likewise for the unlabelled downstream electrodes. **(E)** Injection channel for injection fluid in the T-junction. **(F)** Outport for droplet collection. Scale bar is 1 mm.
